# Seroprevalence of Middle East respiratory syndrome coronavirus (MERS-CoV) in public health workers responding to a MERS outbreak in Seoul, Republic of Korea, in 2015

**DOI:** 10.5365/wpsar.2018.9.3.002

**Published:** 2019-06-06

**Authors:** Boyeong Ryu, Sung-Il Cho, Myoung-don Oh, Jong-Koo Lee, Jaein Lee, Young-Ok Hwang, Jeong-Sun Yang, Sung Soon Kim, Ji Hwan Bang

**Affiliations:** a Seoul Center for Infectious Disease Control and Prevention, Seoul, Republic of Korea.; b Department of Epidemiology, Seoul National University School of Public Health, Seoul, Republic of Korea.; c Department of Internal Medicine, Seoul National University College of Medicine, Seoul, Republic of Korea.; d Department of Family Medicine, Seoul National University College of Medicine, Seoul, Republic of Korea.; e Seoul Metropolitan Government Research Institute of Public Health and Environment, Seoul, Republic of Korea.; f Korea Centers for Disease Control and Prevention, Cheongju, Republic of Korea.; g Division of Infectious Diseases, Seoul Metropolitan Government-Seoul National University Boramae Medical Center, Seoul, Republic of Korea.

The first case of Middle East respiratory syndrome coronavirus (MERS-CoV) in the Republic of Korea was confirmed in May 2015 after a traveller returned from the Middle East. (*1*) There were 186 cases, including 38 deaths, within two months. (*1*) The potential of a single MERS-confirmed patient to result in such a large MERS outbreak constitutes a serious global health concern. (*2*)

During this MERS outbreak, massive public health containment measures were enacted at various levels; these included epidemiological investigations, isolation of suspected and confirmed cases, contact tracing and home quarantine of contacts. Local public health centre (LPHC) and emergency medical services (EMS) personnel responded to the outbreak by conducting initial interviews with suspected cases, transporting patients and specimens and managing contacts. Responders in contact with patients used different levels of personal protective equipment (PPE). Full-protection PPE includes a gown, N95 respirator, gloves and goggles. As the transmissibility of MERS is unclear, (*3*) it is possible that responders were infected by being exposed to MERS patients.

We conducted a cross-sectional study in January 2016 to assess whether LPHC and EMS workers were infected and to determine their degree of exposure. The participants had contact with MERS-confirmed patients or their specimens during the outbreak and volunteered to participate in this study. The survey, which was a face-to-face interview, examined subjects’ general characteristics, professional responsibilities, contact history, symptoms after exposure and use of PPE.

Contact was defined as meeting at least one of the following four criteria: (*4*) being within 2m of a confirmed patient, staying in the same space as a confirmed patient for over 5 minutes, contact with a patient’s respiratory or digestive secretions and contact with specimens from confirmed patients before the sample was packaged. Contact within the same space was graded into four levels according to distance of contact and wearing of PPE. Without full PPE protection: Grade 1 was defined as contact within 2m, and Grade 2 was defined as contact at over 2m. With full PPE protection: Grade 3 was defined as contact within 2m, and Grade 4 was defined as contact at over 2m.

Serum collected from all participants was screened for the presence of MERS-CoV IgG using enzyme-linked immunosorbent assays (ELISAs). One sample with borderline results and five samples with negative ELISA results were retested using indirect immunofluorescence (IIFT) and plaque reduction neutralization (PRNT) tests for confirmation. The indirect ELISA and MERS-CoV IIFT used commercial MERS-CoV IIFT slides (EUROIMMUN, Lübeck, Germany) and followed the manufacturer’s protocol. Analysis was performed using a DE/Axio Imager M1 immunofluorescence microscope (Zeiss, Jena, Germany). The PRNT was performed as previously described. (*5*) The number of plaques per well were counted; reductions in plaque counts of 50% (PRNT50) and 90% (PRNT90) were calculated using the Spearman-Kärber formula. (*5*)

Thirty-four workers participated in the study (**Table 1**): 31 from 11 LPHCs and three from two EMS units. Twenty (58.8%) responders were male; their mean age was 44 (34–56.7) years. Twenty-five participants (73.5%) occupied health-related positions: 11 (32.4%) general health-care staff, 6 (17.6%) nurses, 4 (11.8%) doctors, 3 (8.8%) paramedics and 1 medical laboratory technologist (2.6%). Nine participants (26.5%) were non-health-related workers: 5 (14.7%) technicians, 2 (5.9%) administrators, 1 (2.9%) agricultural worker and 1 (2.9%) unknown.

**Table 1:** Exposure to MERS-confirmed patients (*n* = 34)

Exposure	*n*	(%)
**Grade of contact**		
Grade 1	7	(20.6)
Grade 2	3	(8.8)
Grade 3	20	(58.8)
Grade 4	4	(11.8)
**Longest period of contact**		
< 30 minutes	13	(38.2)
30 minutes to 1 hour	10	(29.4)
1 to 2 hour(s)	6	(17.6)
2 to 5 hours	5	(14.7)
Activity (*n* = 67)*		
Patient transport	24	(35.8)
Patient counselling	10	(14.9)
Ambulance disinfection	10	(14.9)
Specimen transportation	8	(11.9)
Respiratory specimen collection	7	(10.4)
Taking vital signs	4	(6.0)
Discarding exposed goods	3	(4.5)
Other	1	(1.5)
**Symptoms after contact**		
Yes	1	(2.9)
No	33	(97.1)
**PPE education**		
Received	29	(85.3)
Not received	5	(14.7)
**Training in wearing PPE**		
Received	20	(58.8)
Not received	13	(38.2)
Unknown	1	(2.9)

* The 34 study participants performed multiple activities.

PPE: personal protective equipment.

Based on the highest risk contact for each participant, seven (20.6%) of the responders were classified as Grade 1; they were partially protected with at least gloves and an N95 respirator (**Table 1**). They contacted asymptomatic or symptomatic patients, and symptomatic patients wore surgical masks. After MERS-CoV had been confirmed in a patient, all staff were fully protected when in contact with the patient. The closest contact occurred when touching and holding patients during transport. One responder wearing full PPE had a mild fever (37.5 °C) after contact with a symptomatic patient who was later confirmed as infected. Since the response system had not expanded in the early days of the outbreak, she was not tested but was isolated with self-monitoring.

Serum samples were obtained from all 34 participants at an average of 7.3 months (range: 6.7–8.1 months) after exposure. On ELISA, there were 33 (97.1%) negative results and one borderline result. The results of six samples, including one with borderline ELISA results, were negative in the IIFT and PRNT.

In our study, we could not find evidence of MERS infection in the public health providers after direct contact with confirmed patients. This may be because there was a lower risk of transmission when participants were transporting or counselling patients outside of the hospital compared to providing medical assistance within the hospital. In other MERS outbreaks, secondary infections were related to health-care settings. (*1*, *6*) Although the exact route of infection transmission is unknown, aerosolizing procedures in crowded rooms with inadequate infection prevention and control measures were observed in health-care settings. (*7*) In the 2015 Republic of Korea outbreak, some health-care workers without proper PPE were infected in tertiary hospitals, thus emphasizing the optimal use of PPE to prevent MERS infection. (*8*) Moreover, since the participants did not contact any spreaders except one participant who contacted a patient that caused two secondary infections, the risk of transmission from the contacted patients was likely low.

This study had several limitations. First, the survey was conducted 7.3 months after the MERS outbreak, making recall bias possible. Second, it is possible that we missed some mild or asymptomatic cases. Furthermore, because the serological tests were performed several months post-exposure, pre-existing MERS antibodies may have decreased or disappeared in the interval, potentially leading to underestimation. While asymptomatic MERS infection had been detected using RT–PCR testing at the time of outbreak, (*9*) a Saudi Arabian study showed the longevity of MERS-CoV antibodies in MERS patients varied in the severity of illness. For example, antibodies in severely infected patients persisted after 18 months, but milder and subclinical cases detected no antibodies even early on in the disease. (*10*) Third, the number of participants was relatively small and may not be representative or generalizable. Despite these limitations, this study suggests that the risk of MERS transmission to public health professionals responding to MERS outside the hospital setting (i.e. patients’ homes) was low, particularly for those who wore some level of PPE such as masks and gloves. Further study is needed to prospectively survey public health responders including symptomatic or asymptomatic cases to conduct genetic test and serologic test during an outbreak.

In conclusion, the public health providers in our study did not have evidence of MERS transmission after direct contact with confirmed patients when PPE was used properly.

## Ethics

Ethical approval for the study was obtained from the institutional review board of Seoul National University Hospital in Seoul (IRB No. C-1512–049–727).
